# Supplementary material for: Engineering cardiolipin binding to an artificial membrane protein reveals determinants for lipid-mediated stabilization
Source: eLife. 2025 Apr 30;14:RP104237. doi: 10.7554/eLife.104237 (PMC12043315; doi:10.7554/eLife.104237)
Supplement: Supplementary file 1. [file elife-104237-supp1.docx]

**Supporting Information for**

Engineering cardiolipin binding to an artificial membrane protein reveals determinants for lipid-mediated stabilization

Mia L. Abramsson, Robin A. Corey, Jan Škerle, Louise J. Persson, Olivia Andén, Abraham O. Oluwole, Rebecca J. Howard, Erik Lindahl, Carol V. Robinson, Kvido Strisovsky, Erik G. Marklund, David Drew, Phillip J. Stansfeld & Michael Landreh

*Robin A. Corey, Philip J. Stansfeld & Michael Landreh

E-mail: [robin.corey@bristol.ac.uk](mailto:robin.corey@bristol.ac.uk), [phillip.stansfeld@warwick.ac.uk](mailto:phillip.stansfeld@warwick.ac.uk), or [michael.landreh@icm.uu.se](mailto:michael.landreh@icm.uu.se)

**This PDF file includes:**

Tables S1 to S3

Table S1.
Cryo-EM data collection and processing statistics.

| **Data collection and processing** | **ROCKET** | **ROCKET^AAXWA^** |
| --- | --- | --- |
| Magnification | 165,000 | 165,000 |
| Voltage (kV) | 300 | 300 |
| Electron exposure  (e^–^/Å^2^) | 60 | 60 |
| Defocus range (μm) | -0.6 to -1.8 | -0.6 to -1.8 |
| Pixel size (Å) | 0.5076 | 0.5076 |
| Number of images | 10,002 | 10,000 |
| Symmetry imposed | C4 | C4 |
| Particles refined | 98,241 | 142,434 |
| Map resolution (Å)  FSC threshold | 3.77  0.143 | 3.89  0.143 |
| Map sharpening B-factor | -133 | -164 |

Table S2.

Forward and reverse primers for mutagenesis based on the ROCKET sequence.

| **Mutation** | **Primer sequence** (**Forward**/Reverse) | **T_m_ (C°)** |
| --- | --- | --- |
| R9A/K10A/R13A  (AAXWA) | **ctgggcgACCATTATGCTGTTACTG**  GTTTTCTGTGTCTTCTATCAcgccgcta | 57 |
| A61P | **GGTTGTTATTccgCTGTTGCTCAG**  CGAATAGCGGAATTACGA | 60 |
| D7A/S8R | **tcgcAAGATCTGGCGTACCATTATG**  CTATATGTATACAGTTTTCTGTGTCTTcgagc | 62 |

Table S3.

Protein variants; expressed sequence and theoretical molecular weight (MW).

| **Protein** | **Protein sequence (Purification tag)** | **Theoretical MW (Da)** |  |
| --- | --- | --- | --- |
| ROCKET | MSKDTEDSRKIWRTIMLLLVFAILLSAIIWYQITTNPDTSQIATLLSMQLLLIALMLVVIALLLSRQTEQVAESIRRDVSALAYVMLGLLLSLLNRLSLAAEAYKKAIELDPNDALAWLLLGSVLEKLKRLDEAAEAYKKAIELKPNDASAWKELGKVLEKLGRLDEAAEAYKKAIELDPEDAEAWKELGKVLEKLGRLDEAAEAYKKAIELDPNDLEHHHHHH | 25 226.44 |  |
| ROCKET^AAXWA^ | MSKDTEDSAAIWATIMLLLVFAILLSAIIWYQITTNPDTSQIATLLSMQLLLIALMLVVIALLLSRQTEQVAESIRRDVSALAYVMLGLLLSLLNRLSLAAEAYKKAIELDPNDALAWLLLGSVLEKLKRLDEAAEAYKKAIELKPNDASAWKELGKVLEKLGRLDEAAEAYKKAIELDPEDAEAWKELGKVLEKLGRLDEAAEAYKKAIELDPNDLEHHHHHH | 24 999.13 |  |
| ROCKET^A61P^ | MSKDTEDSRKIWRTIMLLLVFAILLSAIIWYQITTNPDTSQIATLLSMQLLLIALMLVVIPLLLSRQTEQVAESIRRDVSALAYVMLGLLLSLLNRLSLAAEAYKKAIELDPNDALAWLLLGSVLEKLKRLDEAAEAYKKAIELKPNDASAWKELGKVLEKLGRLDEAAEAYKKAIELDPEDAEAWKELGKVLEKLGRLDEAAEAYKKAIELDPNDLEHHHHHH | 25 252.48 |  |
| ROCKET^D7A/S8R^ | MSKDTEARRKIWRTIMLLLVFAILLSAIIWYQITTNPDTSQIATLLSMQLLLIALMLVVIALLLSRQTEQVAESIRRDVSALAYVMLGLLLSLLNRLSLAAEAYKKAIELDPNDALAWLLLGSVLEKLKRLDEAAEAYKKAIELKPNDASAWKELGKVLEKLGRLDEAAEAYKKAIELDPEDAEAWKELGKVLEKLGRLDEAAEAYKKAIELDPNDLEHHHHHH | 25 251.54 |  |
| ROCKET^R66A^ | MSKDTEDSRKIWRTIMLLLVFAILLSAIIWYQITTNPDTSQIATLLSMQLLLIALMLVVIALLLSAQTEQVAESIRRDVSALAYVMLGLLLSLLNRLSLAAEAYKKAIELDPNDALAWLLLGSVLEKLKRLDEAAEAYKKAIELKPNDASAWKELGKVLEKLGRLDEAAEAYKKAIELDPEDAEAWKELGKVLEKLGRLDEAAEAYKKAIELDPNDLEHHHHHHHHHH | 25 689.90 |  |
